# Sealing Ability of Polydopamine‐Coated Gutta‐Percha With Bioceramic Sealer: An In Vitro Study

**DOI:** 10.1155/ijod/7270797

**Published:** 2026-06-30

**Authors:** Mariam Fahmy, Aiah A. El-Rashidy, Taheya Moussa

**Affiliations:** ^1^ Biomaterials Department, Faculty of Dentistry, Cairo University, Cairo, Egypt, cu.edu.eg; ^2^ Biomaterials Department, Faculty of Dentistry, The British University in Egypt, Cairo, Egypt, bue.edu.eg

**Keywords:** adaptability, bioceramic sealer, dye penetration, polydopamine, sealing ability, single-cone technique, wetting ability

## Abstract

**Rationale:**

Achieving a three‐dimensional seal of the root canal system is essential for successful endodontic treatment. However, the hydrophobic nature of gutta‐percha may compromise the interaction with the hydrophilic bioceramic sealers, leading to interfacial gaps and potential microleakage. Polydopamine (PDA) surface modification has recently emerged as a bioinspired strategy to enhance surface wettability and adhesion of biomaterials.

**Aim:**

To evaluate the sealing ability, adaptability, and wetting ability at the sealer–gutta‐percha interface of PDA‐coated gutta‐percha cones with bioceramic sealer using the single‐cone obturation technique.

**Methods:**

Sealing ability was assessed by measuring the apical dye penetration. Fourteen extracted mandibular premolars were instrumented using ProTaper Next rotary system, then randomly assigned into two groups (*n* = 7): uncoated or PDA‐coated gutta‐percha with bioceramic sealer. Interfacial adaptability between sealer and gutta‐percha was examined using scanning electron microscopy (SEM). Wetting ability was evaluated by measuring the contact angle between the bioceramic sealer and the gutta‐percha discs using 28 gutta‐percha discs (*n* = 14) for both groups. Data were analyzed using one‐way ANOVA and Student’s *t*‐test (*α* = 0.05).

**Results:**

In the sealing ability test, PDA‐coated gutta‐percha demonstrated significantly lower dye penetration (3.04 ± 0.59 mm) than the uncoated gutta‐percha (4.3 ± 1.11 mm) (*p* < 0.05). SEM analysis demonstrated improved interfacial adaptability between the bioceramic sealer and PDA‐coated gutta‐percha, with no detectable gaps after 28 days in simulated body fluid (SBF), compared to uncoated gutta‐percha, which showed interfacial gaps throughout the root at all time points. In addition, PDA‐coated gutta‐percha significantly reduced the contact angle (28.8° ± 9.4°) compared to the uncoated gutta‐percha discs (77.4° ± 27.1°) (*p* < 0.05), indicating enhanced wettability.

**Conclusion:**

PDA surface modification significantly improved the wettability, interfacial adaptation, and the sealing performance of the gutta‐percha when used with a bioceramic sealer. Clinically, this bioinspired coating may contribute to the quality and durability of root canal obturation.

## 1. Introduction

The success of root canal treatment is achieved through the triad of thorough mechanical preparation and canal debridement, effective disinfection, and adequate obturation of the canal space. Gutta‐percha remains the gold standard root canal filling material owing to its biocompatibility, nonstaining nature, and radiopacity. However, gutta‐percha does not chemically bond to root canal sealers, which may result in microgap formation, particularly if the sealer undergoes setting shrinkage. These limitations are further exacerbated by the inherent hydrophobicity of gutta‐percha, particularly when used with the single‐cone technique, in which sealing largely depends on the sealer’s interfacial adaptation. Consequently, microleakage may occur along the gutta‐percha–sealer interface [1–4].

Bioceramic sealers have increasingly replaced conventional sealers due to their ability to chemically bond to dentin through hydroxyapatite formation, hydrophilicity, and alkaline nature, thereby reducing microleakage and improving adaptability [5]. Although the adaptability and sealing performance of bioceramic sealers to root dentin have been extensively investigated [6–8], the gutta‐percha–sealer interface continues to be a structurally weak component of the obturation complex [9].

Gutta‐percha’s surface modification is considered a potential strategy to optimize its sealing ability and enhance the bioceramic sealer’s wettability [10]. Several gutta‐percha surface modifications have been proposed to reinforce the core–sealer interface and improve the sealing ability. Bioactive coatings that mimic the surface chemical composition of dentin and sealer aim to enhance chemical and mechanical bonding. However, commercially available coated gutta‐percha cones, including methacrylate‐resin‐based, bioceramic‐coated, and glass‐ionomer‐coated systems, have not consistently achieved a complete and durable seal [11].

Alternative approaches have attempted to create a bioactive monoblock system by impregnating gutta‐percha with bioactive coatings, for example, bioceramic‐coated gutta‐percha. However, commercially available bioactive‐coated gutta‐percha cones demonstrate compromised sealing due to the inhomogeneous coating distribution along the gutta‐percha surface [12].

Polydopamine (PDA), inspired by the adhesive mechanisms of mussels, is derived from the oxidative polymerization of dopamine. It exhibits strong wet adhesion and surface functionalization across a wide range of substrates. PDA has emerged as an innovative coating material capable of enhancing surface hydrophilicity, chemical reactivity, biocompatibility, and adhesiveness through biosurface functionalization [13]. Accordingly, PDA represents a promising surface modification strategy to enhance the interaction between gutta‐percha cones and bioceramic sealers. PDA can adhere to the gutta‐percha surface via physical adsorption and covalent interactions, forming a stable, functionalized layer rich in catechol groups. In addition, this bioactive interphase facilitates interaction with calcium ions released from the bioceramic sealers, thereby enhancing the chemical affinity between the bioceramic sealer and the gutta‐percha cone, which could aid in creating a monoblock and reduce the risk of microleakage [14].

Therefore, the aim of this study was to evaluate the sealing ability, adaptability, and wetting ability of PDA‐coated gutta‐percha compared to an uncoated gutta‐percha cone used with a bioceramic sealer in the obturated root canals using the single‐cone technique. The null hypothesis was that there would be no difference in sealing ability, adaptability, and wetting ability between PDA‐coated and uncoated gutta‐percha used with a bioceramic sealer, using the single‐cone technique in the obturated root canals.

## 2. Materials and Methods

### 2.1. Material

Dopamine hydrochloride was purchased from Sigma–Aldrich (Darmstadt, Germany) and dissolved in TRIS buffer (Biodiagnostics, Dokki, Egypt). Gutta‐percha single cones (Conform Fit) were used for obturation, matched to the ProTaper Next file size X3 (Dentsply Sirona, USA). The bioceramic sealer used was CeraSeal (Meta Biomed, Cheongju, Korea), a premixed calcium silicate‐based root canal sealer. Simulated body fluid (SBF) was laboratory‐prepared (Faculty of Pharmacy, Cairo University), following standard formulation protocols. Additionally, an aqueous solution of 1% methylene blue dye (powder purchased from Sigma–Aldrich, Darmstadt, Germany) was prepared in the laboratory (Department of General Pathology, Faculty of Medicine, Cairo University) before use.

### 2.2. Sample Size Calculation

Sample size calculations were performed using PS: Power and Sample Size Program (version 3.1.2; Vanderbilt University, Nashville, TN, USA) [15], assuming an independent Student’s *t*‐test with a significance level of 0.05 and a statistical power of 80%.

Based on previous studies, a minimum of three samples per group was required for the sealing ability test [16] and five samples per group for the wetting ability test [17]. To compensate for potential dropouts and to enhance statistical validity and reliability, the final sample sizes were increased to 7 and 14 per group, respectively.

### 2.3. Study Design and Grouping of Specimens

PDA‐coated gutta‐percha surfaces were prepared via 24 and 72 h dip‐coating. Characterization of chemical, morphological, and bioactivity was conducted on three gutta‐percha cones per experimental group. To characterize the chemical composition and surface morphology, a total of nine gutta‐percha cones were allocated to the groups (uncoated, 24 h, and 72 h PDA‐coated). In the bioactivity characterization, a total of six gutta‐percha groups were allocated (uncoated and 72 h PDA‐coated cones). Based on these characterization results, the most suitable dip‐coating duration (72 h) was selected for the study.

**Figure 1:**
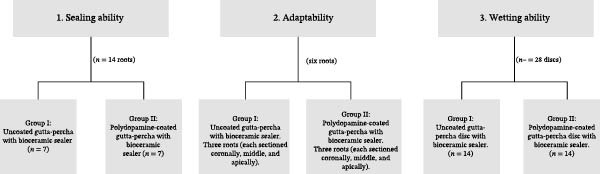
Flow diagram illustrating sample allocation for sealing ability, adaptability, and wetting ability tests.

For the evaluation of the uncoated and PDA‐coated gutta‐percha with the bioceramic sealer, a total of 20 obturated root canals were allocated to two experimental tests: sealing ability (14 roots; *n* = 7) and adaptability (six roots; three per group). About 28 disc‐shaped specimens were allocated to the wetting ability test, as illustrated in Figure 1.

For the sealing ability test, 14 decoronated obturated roots were randomly and equally distributed into two groups: group I: uncoated gutta‐percha with a bioceramic sealer and group II: PDA‐coated gutta‐percha with a bioceramic sealer (*n* = 7).

Regarding the adaptability evaluation, interfacial gaps between the gutta‐percha cone and sealer were measured (µm) using environmental scanning electron microscope (ESEM). Six decoronated, obturated roots were randomly and equally distributed among the tested groups: uncoated and PDA‐coated gutta‐percha with a bioceramic sealer (three roots per group). At each time point (baseline at day 0 and after 14 and 28 days of immersion in SBF), each obturated root was transversely sectioned at three levels (coronal, middle, and apical), yielding nine specimens per group. Therefore, each time interval contained three specimens.

Finally, to evaluate the wetting ability of the bioceramic sealer on the gutta‐percha surface, 28 disc‐shaped specimens were randomly assigned to the two test groups: uncoated and PDA‐coated gutta‐percha (*n* = 14).

The overall specimen distribution across the three experimental tests is summarized in Table 1.

**Table 1:** Sample distribution across experimental tests.

Testing procedure	Number of specimens per test		
	Group I: uncoated gutta‐percha	Group II: PDA‐coated gutta‐percha	Total
Sealing abillity (dye penetration)			
After 14 days of immersion in SBF	7 obturated root specimens	7 obturated root specimens	14
Adaptability (SEM)			
Baseline	3 specimens	3 specimens	18
After 14 days of immersion in SBF	3 specimens	3 specimens	
After 28 days of immersion in SBF	3 specimens	3 specimens	
Wetting ability (contact angle)			
Immediately	14 discs	14 discs	28
Total number of specimens per group	30	30	Total number = 60

### 2.4. Preparation of PDA Solution and Coating Procedure

#### 2.4.1. Preparation of PDA Solution

A PDA solution was prepared to deposit a mussel‐inspired PDA layer on the surfaces of the gutta‐percha cones. A diluted aqueous dopamine solution was prepared by dissolving 2 mg/mL of dopamine hydrochloride powder (Sigma–Aldrich, Germany) in a 10 mM TRIS‐HCL buffer (pH 8.5) (Biodiagnostics, Egypt) following the protocol outlined by Lee et al. [18].

#### 2.4.2. Gutta‐Percha Coating Procedure

The single gutta‐percha cones (Conform Fit, Dentsply Sirona, USA) matched to the ProTaper Next X3 file were first abraded with a #1000 SiC paper (Struers, Denmark) and subsequently washed with acetone, ethanol, and deionized water in an ultrasonic cleaner (MCS, AS ONE Corporation, Osaka, Japan) for 5 min and then dried at 37°C in an incubator (Binder BD56, Germany) for 1 h. Gutta‐percha cones were coated with PDA by dip‐coating for 24 and 72 h at 37°C. Dopamine self‐polymerization is accompanied by a color change from colorless to pale brown, then to deep brown as deposition proceeds. After the incubation period, PDA‐coated gutta‐percha cones were rinsed with deionized water and air‐dried [19, 20].

### 2.5. Characterization of the Uncoated and the PDA‐Coated Gutta‐Percha Cones

#### 2.5.1. Chemical Characterization

Chemical characterization of the uncoated and PDA‐coated gutta‐percha (24 and 72 h) cones was performed using attenuated total reflectance Fourier transform infrared spectroscopy (ATR‐FTIR) with a Nicolet iS50 ATR spectrometer (Thermo Scientific, USA). The ATR‐FTIR spectra were used as indicators of the deposition of PDA functional groups on gutta‐percha cones upon immersion in the dopamine solution for 24 and 72 h [21].

#### 2.5.2. Morphological Characterization

The surface morphology of the uncoated and PDA‐coated gutta‐percha (24‐ and 72 h) cones was qualitatively assessed using ESEM (FEI Quanta 3D 200i, Thermo Fisher Scientific). Coating uniformity of the PDA layer after dip coating was evaluated by visual inspection of the micrographs for surface roughness, homogeneity, and coating continuity, following the descriptive criteria outlined by Ryu et al. [22]. Following characterization, the 72 h dip‐coated PDA gutta‐percha cones exhibited more favorable surface and chemical properties and were therefore selected for all subsequent experimental evaluations.

#### 2.5.3. Bioactivity Characterization

SBF was laboratory‐prepared (Faculty of Pharmacy, Cairo University) according to the protocol described by Kokubo and Takadama and ISO 23317:2012 [23, 24]. The bioactivity of the uncoated and PDA‐coated gutta‐percha cones after 14 days of immersion in SBF was evaluated using ESEM (FEI Quanta 3D 200i, Thermo Fisher Scientific) to assess the surface morphology. The elemental composition of the surface layer upon immersion in SBF was further analyzed using energy dispersive X ray (EDX) spectroscopy.

### 2.6. Sealing Ability Evaluation

#### 2.6.1. Teeth Selection and Preparation

Fourteen freshly extracted mandibular second premolars with sound roots were selected. Teeth extracted for periodontal, prosthetic, or orthodontic reasons at the Oral and Maxillofacial Surgery Department, Faculty of Dentistry, Cairo University were used. Teeth were used according to the approval of the ethical committee of the Faculty of Dentistry, Cairo University (Number 4‐9‐23). Following extraction, the teeth were cleaned of adherent soft tissues and calculus and stored in 0.9% physiological saline at 4°C to maintain dentin hydration. Buccolingual and mesiodistal radiographs were obtained for the collected teeth, and teeth exhibiting previous endodontic treatment, root resorption, an open apex, or severe root curvature were excluded. Only teeth with a fully formed apex and a single straight root canal were included. To standardize the roots’ length, the roots’ surfaces were cleaned with manual scalers, and each tooth was decoronated 14 mm from the apex below the cementoenamel junction using a double‐sided diamond disc (DFS Diamon‐Freudensprung‐Schmiede, Germany) mounted on a low‐speed straight handpiece (MCS TX‐B, COXO, China) under constant water cooling.

Randomization of the decoronated roots was performed using a computer‐generated random number sequence generator (Excel, Microsoft, USA), which allocated the specimens into two groups: group I (uncoated gutta‐percha) and group II (PDA‐coated gutta‐percha). Because the uncoated and PDA‐coated gutta‐percha cones differed in color, operator blinding during obturation was not feasible. However, to minimize assessment bias, all specimens were coded after obturation, and the examiner performing the microleakage analysis was fully blinded to group allocation throughout the evaluation.

#### 2.6.2. Root Canal Preparation

The canal patency of the decoronated roots was verified using a #10 K‐file (Dentsply Malliefer, Ballaigues, Switzerland). A #15 K‐file with a rubber stopper was then introduced into the canal until its tip became visible at the apical foramen. The file length was measured from the tip to the stopper, and the working length was set 1 mm short of this measurement; this value was recorded as the actual working length. A glide path was established using manual K‐files up to size #20. Canal orifices were preflared with an SX ProTaper file (Dentsply Sirona, USA), followed by shaping the canals with ProTaper Next files from X1 to X3 (Dentsply Sirona, USA), reaching the full working length. Instrumentation was performed according to the manufacturer’s recommended speed and torque settings. During instrumentation, canals were constantly irrigated alternatively with 5 mL of 5.25% sodium hypochlorite (NaOCl) using a 27‐gauge needle (Splash, Fanta Dental Co., Korea) and 19% ethylenediaminetetraacetic acid (EDTA) gel (MD‐ChelCream, META Biomed, Korea) to effectively remove both organic and inorganic components of the smear layer [25]. A final rinse with saline was performed to eliminate any residual NaOCl. The canals were then dried using absorbent paper points (Meta Biomed, Korea).

#### 2.6.3. Samples Grouping and Root Canal Obturation

Roots were randomly allocated to receive either obturating materials (*n* = 7), group I: uncoated gutta‐percha cone with CeraSeal bioceramic sealer (Meta Biomed, Cheongju, Korea), or group II: PDA‐coated gutta‐percha cone with the same bioceramic sealer. Each root was placed flush with its apex on a glass slab, and the gutta‐percha single cone X3 (30/0.07) (Conform Fit, Dentsply Sirona, USA) was introduced into the canal.

After root canals were dried and master cones were selected, a small portion of the bioceramic sealer (CeraSeal Bioceramic, Meta Biomed, Korea) was injected into the prepared canal using a fine‐tip sealer syringe, not deeper than the coronal half of the canal length. The master cone tip was covered with a sealer and inserted to the working length. A heated endodontic plugger was then used to remove the excess gutta‐percha. Following obturation in both groups, the canal orifices were sealed with a zinc oxide‐based temporary restorative material (Cavit G, 3M ESPE, St. Paul, MN, USA). The obturated roots were stored at 37°C in 100% humidity for 1 week to allow the complete setting of the bioceramic sealer. All obturations were performed by the same experienced operator using a consistent obturation technique to minimize variability in pressure and sealer application.

#### 2.6.4. Dye Penetration Test

After storage, the sealing ability of the tested groups (*n* = 7) was assessed using a linear dye‐penetration test. Obturated roots were immersed in SBF for 14 days, with the solution refreshed every 2 days; thereafter, a dye penetration test was performed to assess apical leakage [26]. The obturated roots were rinsed with deionized water, air‐dried, and coated with two layers of nail varnish (YOLO Cosmetics, Egypt), leaving the apical 3 mm exposed to allow dye penetration only through the root apex. The obturated roots were then immersed in 1% methylene blue dye (Faculty of Dentistry, Cairo University) and stored at 37°C. After 48 h, the obturated roots were thoroughly rinsed under running water for 5 min. The nail varnish was then removed using manual scalers, and the teeth were allowed to dry. The obturated roots were embedded in an acrylic block and then sectioned buccolingually in the longitudinal direction using an automated diamond saw (Isomet 4000, Buehler) with copious coolant irrigation. Only half of each sample was examined, and a single measurement was recorded per specimen.

Apical leakage was determined by measuring the distance from the root apex to the most coronal point at which the dye no longer penetrated the filling materials. Images were captured under a stereomicroscope (Olympus VM‐ILA‐2, Japan). Approximately 30x magnification and exposure settings were standardized across groups. The readings were measured in micrometers and converted into millimeters using ImageView Analysis software (ToupTek Photonics, Zhejiang, China) [27]. Each of the seven sectioned samples per group was analyzed individually; only half of each sample was examined, and a single measurement was recorded per specimen.

### 2.7. Adaptability Evaluation

**Figure 2:**
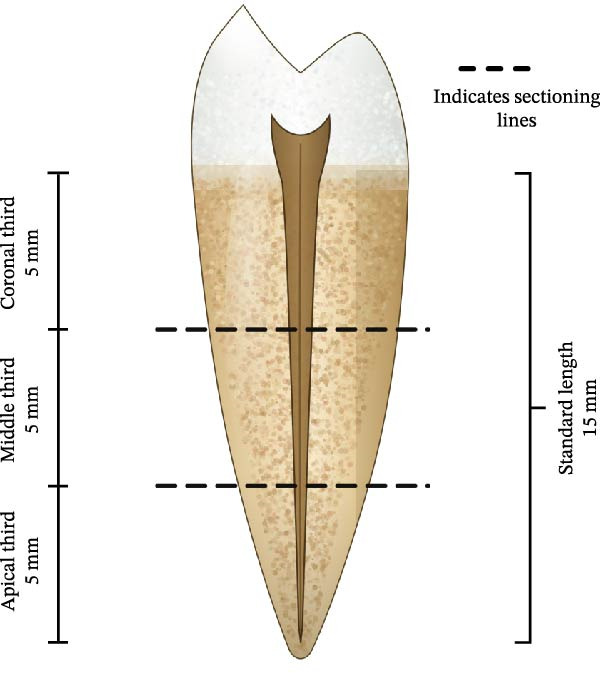
Schematic illustration of the transverse sections’ measurement used for the adaptability test.

To evaluate adaptability, interfacial gaps between the gutta‐percha cone and sealer were measured (µm). The six decoronated, obturated roots were randomly and equally distributed among the tested groups: uncoated and PDA‐coated gutta‐percha with a bioceramic sealer (three roots per group). Each group was further subdivided into three subgroups (three sectioned specimens per subgroup) based on time points (baseline at day 0 and after 14 and 28 days of immersion in SBF). Each obturated root was embedded in an acrylic block to facilitate precise handling during sectioning. Each root was transversely sectioned at 3 mm (apical third), 6 mm (middle third), and 9 mm (coronal third) from the established working length, as shown in Figure 2, using an automated diamond saw (Isomet 4000, Buehler) with copious coolant irrigation. At each level, two representative images from the transverse sections were acquired using ESEM (FEI Quanta 3D 200i, USA), and one image from each section was selected for analysis. For each sectioned level, three interfacial gap measurements (µm) were obtained. Their mean value was calculated and used as a representative descriptive measure for adaptability assessment. To prevent any interoperator variability, ESEM photomicrographs of all sections were analyzed by a single operator. Gap distances (µm), if any, were recorded using ImageJ software (National Institutes of Health, USA). To assess measurement reliability, 20% of the images were remeasured twice by the same examiner after 2 weeks (intraexaminer reliability) and independently by a second calibrated examiner (interexaminer reliability).

### 2.8. Wetting Ability Evaluation

#### 2.8.1. Sample Preparation

Gutta‐percha discs were prepared by dispensing thermoplasticized gutta‐percha pellets heated to 200°C into Teflon molds (10 mm × 1 mm) placed on a clean glass slab using an obturation gun (Fast Fill, Eightheeth, China) equipped with a #23‐gauge needle (Fast Fill, Eighteeth, China). Upon reaching the target temperature, the activation cuff of the flow handpiece was pressed to extrude gutta‐percha onto a glass plate. The extruded gutta‐percha was flattened between two clean glass slabs to produce standardized disc‐shaped gutta‐percha specimens with smooth and flat surfaces. A total of 28 discs were prepared and allocated into the two experimental groups: group I (uncoated gutta‐percha) discs and group II (PDA‐coated gutta‐percha) discs (*n* = 14). The discs were subsequently mounted on a plain glass surface and analyzed using an optical tensiometer (Theta Flow, Attension, Biolin Scientific, Sweden). At room temperature, the volume of a sessile droplet from the bioceramic sealer was adjusted with a calibrated insulin syringe (1 µL graduations) positioned perpendicular to the disc surface at a fixed height (~1–2 mm). Contact angle measurements were performed in triplicate for each specimen, and the mean was calculated for each sample. The sealer drop profile was calculated using OneAttension software (Biolin Scientific AB, Gothenburg, Sweden). Subsequently, the height (*h*) and base width (*b*) of each droplet were measured using an optical tensiometer. These measurements were used to calculate the contact angles based on the spherical cap model equation (*θ* = 2.arc [cos *h*/*b*]) [28].

#### 2.8.2. Statistical Analysis

Data management and statistical analysis were conducted using the Statistical Package for Social Sciences SPSS Statistics version 20 (SPSS Inc., Chicago, IL, USA). Dye penetration depth (mm) and contact angle values (°) were expressed as the mean and standard deviation (SD). Data normality was assessed using the Kolmogorov–Smirnov and Shapiro–Wilk tests. For normally distributed data, comparisons between groups were performed using the independent Student *t*‐test. All *p*‐values were two‐sided, and *p*‐values ≤0.05 were considered statistically significant.

## 3. Results

### 3.1. Characterization of the Gutta‐Percha Cones

#### 3.1.1. Chemical Characterization

**Figure 3:**
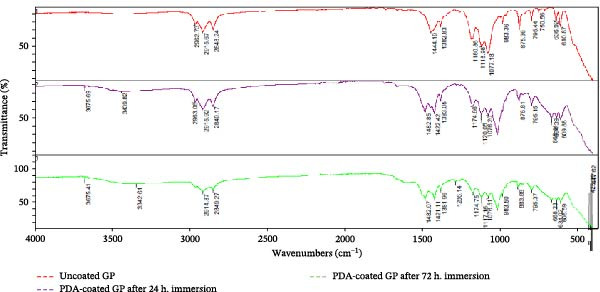
Attenuated total reflectance Fourier transform infrared spectroscopy (ATR‐FTIR) spectra of uncoated gutta‐percha and polydopamine (PDA)‐coated gutta‐percha after 24 and 72 h immersion in dopamine solution.

The ATR‐FTIR spectra of the investigated groups are shown in Figure 3. The ATR‐FTIR spectrum of the uncoated gutta‐percha exhibited characteristic absorption peaks at 2950–2850 cm^−1^ corresponding to C─H stretching, 1452–1444 cm^−1^ for CH_2_ scissoring, 1380 cm^−1^ for CH_3_ bending, 1238–1239 cm^−1^ for C─C stretching, 1076 cm^−1^ for C─O or C─C skeletal stretching, and 900–750 cm^−1^ for C─H out‐of‐plane bending (trans‐alkene). These features are indicative of trans‐1,4‐polyisoprene, the main structural component of gutta‐percha. In contrast, the ATR‐FTIR spectrum of the PDA‐coated gutta‐percha displayed additional absorption bands at 3400–3700 cm^−1^, attributed to broad O─H and N─H group interactions (hydroxyl and amine groups) characteristic of the catechol moieties in PDA and a peak at 1480 cm^−1^ corresponding to C ═ C stretching within the aromatic ring of catechol. These PDA‐related peaks were evident at both 24 and 72 h coating durations. The increased intensity of PDA‐related peaks after 72 h of immersion confirmed successful PDA deposition and the formation of a thicker, denser coating layer on the gutta‐percha surface.

#### 3.1.2. Morphological Characterization

**Figure 4:**
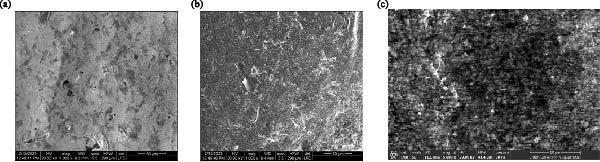
Environmental scanning electron microscope (ESEM) images of gutta‐percha cones. (a) Uncoated gutta‐percha cone, at 1000x magnification. (b) Polydopamine (PDA)‐coated gutta‐percha cone after 24 h of immersion in dopamine solution, at 1000x magnification. (c) Polydopamine (PDA)‐coated gutta‐percha cone after 72 h of immersion in dopamine solution, at 5000x magnification. Scale bar 50 µm.

Figure 4 presents ESEM images of the gutta‐percha surface before and after PDA coating at 24 and 72 h (magnification 1000x) to assess coating uniformity. In Figure 4a, the uncoated gutta‐percha surface appears bright, indicating the absence of a PDA layer. In Figure 4b, the surface appears darker, suggesting initial PDA deposition after 24 h. After 72 h of immersion (Figure 4c), the surface exhibits uniform darkening at a higher magnification (5000x), confirming the formation of a continuous and homogeneous PDA layer.

**Figure 5:**
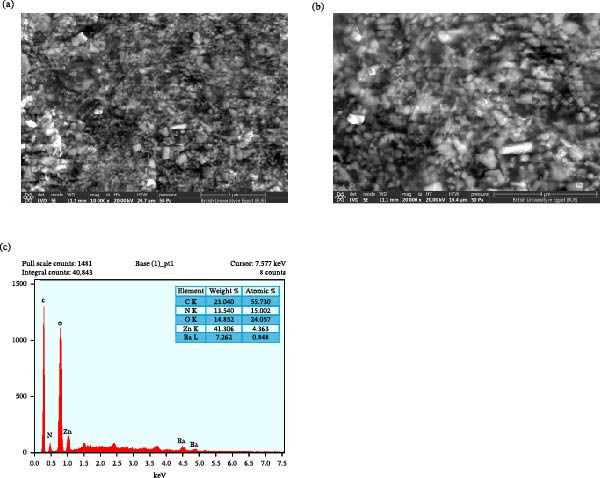
Environmental Scanning electron microscope (ESEM) images and energy dispersive X‐ray spectroscopy (EDX) analysis of polydopamine (PDA)‐coated gutta‐percha cone after 72 h of dip‐coating in dopamine solution and before immersion in simulated body fluid. (a) SEM image at 10,000x magnification, scale bar 5 µm. (b) SEM image at 20,000x magnification, scale bar 4 µm. (c) EDX spectrum for polydopamine (PDA)‐coated gutta‐percha cone after 72 h of dip coating and before immersion in simulated body fluid.

Figure 5 displays a uniform PDA coating on the gutta‐percha surface following 72 h of dip‐coating in dopamine solution (magnification 10,000x) (Figure 5a). At higher magnification (20,000x) (Figure 5b), the PDA‐coated gutta‐percha exhibits a fine‐grained or nodular surface morphology characteristic of PDA aggregates. EDX analysis (Figure 5c) revealed carbon (55%), oxygen (24%), and nitrogen (15%), confirming the expected chemical composition of the PDA layer. Signals for zinc (4%) and barium (0.8%) intrinsic to the underlying gutta‐percha matrix were also detected, likely due to the relatively thin coating (~50–200 nm).

Based on the characterization results, gutta‐percha cones coated with PDA for 72 h exhibited enhanced chemical and surface properties and were therefore selected for further experimental evaluation.

#### 3.1.3. Bioactivity Characterization

**Figure 6:**
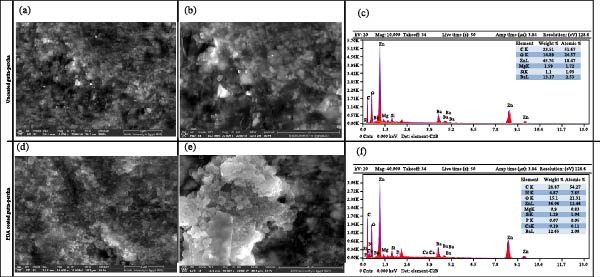
Environmental Scanning electron microscope (ESEM) images and energy dispersive X‐ray spectroscopy (EDX) of an uncoated gutta‐percha cone and a polydopamine (PDA)‐coated gutta‐percha cone after immersion in simulated body fluid for 14 days. (a) SEM image of uncoated gutta‐percha cone at 10,000x magnification, scale bar 5 µm, and (b) at 20,000x magnification, scale bar 4 µm. (c) EDX spectrum of an uncoated gutta‐percha cone. (d) SEM image of polydopamine (PDA)‐coated gutta‐percha at 10,000x magnification, scale bar 5 µm. (e) SEM image at 20,000x magnification, scale bar 4 µm. (f) EDX spectra of polydopamine (PDA)‐coated gutta‐percha cones.

ESEM images of the uncoated gutta‐percha cones after 14 days of immersion in SBF (Figure 6a,b) revealed scattered granules or clusters across the surface. These bright spots represent filler particles such as zinc oxide or barium sulfate embedded in the polyisoprene matrix. EDX analysis (Figure 6c) confirms the presence of zinc (18.47%) and barium (2.53%), in addition to carbon (51.67%), which is the primary component of trans‐1,4‐polyisoprene (hydrocarbon polymer).

Conversely, ESEM images of PDA‐coated gutta‐percha after 14 days of immersion in SBF (Figure 6d,e) displayed hemispherical globules and flake‐like crystal structures on the surface at magnifications of 10,000x and 20,000x, respectively. EDX analysis (Figure 6f) revealed calcium (0.11%) and phosphorus (0.05%), indicating the formation of calcium phosphate‐like deposits, suggesting enhanced bioactivity and surface mineralization of the PDA‐coated gutta‐percha.

### 3.2. Sealing Ability

**Figure 7:**
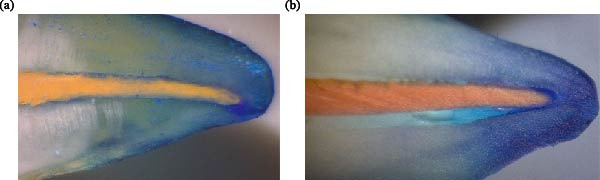
Stereomicroscope images at 30x magnification showing dye penetration in obturated roots. (a) Uncoated gutta‐percha cone with bioceramic sealer. (b) Polydopamine (PDA)‐coated gutta‐percha cone with bioceramic sealer.

The homogeneity and normality assumptions for the dye penetration data were met. PDA‐coated gutta‐percha showed significantly lower dye penetration (3.04 ± 0.59 mm) than uncoated gutta‐percha (4.30 ± 1.11 mm) (*p* = 0.041), using the same bioceramic sealer, Table 2. Representative stereomicroscopic images illustrating the extent of the dye penetration in the longitudinally sectioned samples from both groups at 30x magnification are shown in Figure 7.

**Table 2:** Mean and SD values of apical dye penetration (mm).

Groups	Mean (mm)	SD (mm)	*p*‐Value
Uncoated gutta‐percha group	4.30	1.11	0.041 ^∗^
PDA‐coated gutta‐percha group	3.04	0.59	

^∗^Significant, significance level *p* ≤ 0.05.

### 3.3. Adaptability

**Figure 8:**
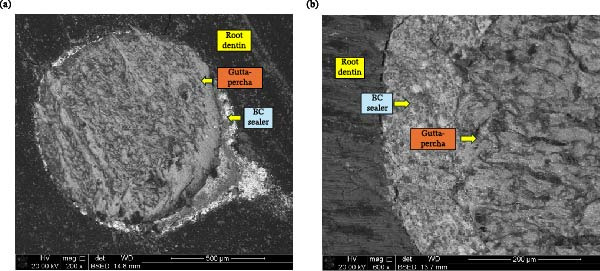
Representative environmental scanning electron microscope (ESEM) images for polydopamine (PDA)‐coated gutta‐percha cone adaptability at (a) 200x magnification, scale bar 500 µm and (b) at 600x magnification, scale bar 200 µm.

ESEM revealed differences in interfacial adaptation between the groups across all root regions (apical, middle, and coronal) and at all time points. The sealer–gutta‐percha interface was evaluated at both low and high magnifications to assess sealer distribution and the presence of interfacial gaps (Figure 8). Due to the limited sample size, no inferential statistics were applied.

**Figure 9:**
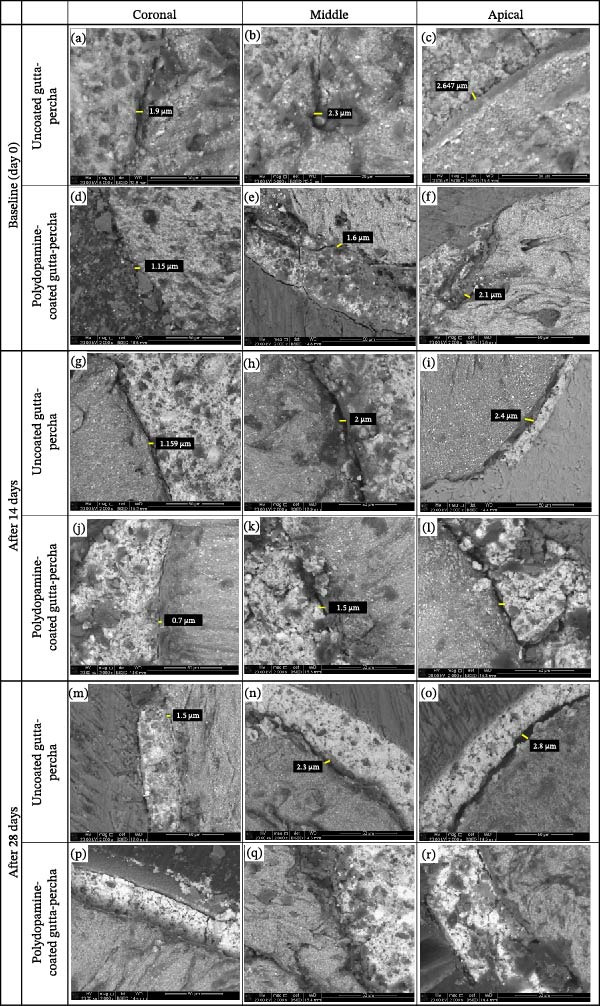
Environmental Scanning electron microscope (ESEM) images show the interface between an uncoated and a polydopamine (PDA)‐coated gutta‐percha cone with a bioceramic sealer. (a) At baseline (day 0), showing the interface of an uncoated gutta‐percha cone at the coronal third, (b) middle third, and (c) apical third at 5000x magnification, scale bar 50 µm. (d) At baseline (day 0), showing the interface of polydopamine (PDA)‐coated gutta‐percha cone at the coronal third, (e) middle third, and (f) apical thirds at 2000x magnification, scale bar 50 µm. (g) At day 14, the interface of the uncoated cone at the coronal third, (h) middle third, and (i) apical third. (j) At day 14, the interface of the polydopamine (PDA)‐coated cone at the coronal third, (k) middle third, and (l) apical thirds, at magnification 2000x, scale bar 50 µm. (m) At day 28, the interface of the uncoated cone at the coronal, (n) middle, and (o) apical thirds. (p) At day 28, the interface of polydopamine (PDA)‐coated cone at the coronal, (q) middle, and (r) apical thirds, at 2000x magnification, scale bar 50 µm.

At baseline (day 0), specimens obturated with uncoated gutta‐percha exhibited poor marginal adaptation to the bioceramic sealer with interfacial gaps present in all root thirds, most pronounced in the apical region (2.64 µm), while the smallest interfacial gap was detected in the coronal third (1.9 µm) (Figure 9a–c). In contrast, specimens obturated with PDA‐coated gutta‐percha demonstrated improved marginal adaptation to the sealer with smaller interfacial gaps, particularly in the coronal and middle thirds (1.5 and 1.6 µm, respectively), whereas the apical third (2.1 µm) still showed the largest gap, albeit smaller than that in the uncoated group (Figure 9d–f).

After 14 days of immersion in SBF, specimens obturated with uncoated gutta‐percha showed increased interfacial gap size with the bioceramic sealer from the coronal third (1.59 µm), enlarging in the middle third (2 µm), and reaching their maximum in the apical third (2.4 µm) (Figure 9g–i). Conversely, the PDA‐coated gutta‐percha group maintained better adaptation, with comparatively smaller gaps across all root regions increasing in size from the coronal third (0.7 µm) to a moderate gap in the middle third (1.3 µm), while the apical region displayed the largest gaps (2 µm), compared to the uncoated group (Figure 9j–l).

After 28 days of immersion in SBF, specimens obturated with the uncoated gutta‐percha continued to exhibit visible interfacial gaps along the entire root length at the sealer–gutta‐percha interface. The gaps were smallest in the coronal region (1.5 µm), larger in the middle third (2.3 µm), and most prominent in the apical third (2.8 µm) (Figure 9m–o). In contrast, specimens obturated with PDA‐coated gutta‐percha demonstrated marked improvement in interfacial adaptation, with no detectable gaps in any root region, indicating a time‐dependent enhancement in adaptability (Figure 9p–r).

### 3.4. Wetting Ability

**Figure 10:**
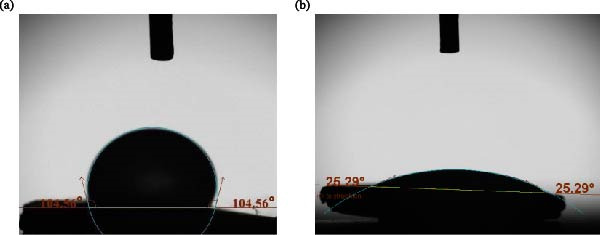
Contact angle formed by bioceramic sealer and (a) uncoated gutta‐percha disc and (b) polydopamine (PDA)‐coated gutta‐percha disc.

The homogeneity and normality assumptions were met for the contact angle data. The contact angle between the PDA‐coated gutta‐percha discs and bioceramic sealer was significantly lower (28.75° ± 9.42°) than that between the uncoated gutta‐percha discs and the bioceramic sealer (77.42° ± 27.10°) (*p* = 0.000001). Table 3 shows the results. Representative contact angle images are shown in Figure 10.

**Table 3:** Mean and SD values of contact angle (°).

Groups	Mean (°)	SD (°)	*p*‐Value
Uncoated gutta‐percha group	77.42	27.10	0.0001 ^∗^
PDA‐coated gutta‐percha group	28.75	9.42	

^∗^Significant, significance level *p* ≤ 0.05.

## 4. Discussion

Achieving three‐dimensional chemicomechanical preparation and obturation is essential to limit microbial leakage along the root canal system. This is typically accomplished by using a gutta‐percha core in conjunction with an endodontic sealer. However, marginal gaps at dentin–sealer and core–sealer interfaces can compromise the success of root canal treatment. In line with this endodontic objective, the present study aimed to evaluate the sealing ability, adaptability, and wetting ability of uncoated and PDA‐coated gutta‐percha, both used with a bioceramic sealer, using the single‐cone obturation technique.

Conventional gutta‐percha has been the material of choice for root canal obturation due to its inert nature. However, its primary limitations include limited adhesion to the root dentin and sealers, necessitating a fluid‐tight sealer to fill gaps between the root dentin and the obturation material. Over time, microleakage can occur, particularly if the sealer shrinks or dissolves.

PDA coating represents a promising surface functionalization for various substrates. The strong adhesion of PDA is primarily due to its catechol and amine groups, which enable it to bind effectively to both hydrophilic and hydrophobic substrates, thereby enhancing their hydrophilicity [29]. In a study by Park and Jung [30], the surface of an intervertebral fusion implant made of polyether ether ketone (PEEK) was modified with several coatings, including polyethylene glycol, hyaluronic acid, and PDA, to enhance its hydrophilicity and biocompatibility. PDA significantly increased the surface hydrophilicity compared to that of the other groups. Another study by Yu et al. [31] demonstrated a substantial reduction in the water contact angle of untreated zirconia (Y‐TZP) after PDA–zirconia surface treatment. Accordingly, PDA was used in the present study to coat hydrophobic gutta‐percha cones, thereby transforming their surfaces into bioactive, hydrophilic ones, particularly when applied with sealers during endodontic obturation.

In the present study, CeraSeal (Meta Biomed., Korea), a hydraulic calcium silicate‐based sealer, was used. CeraSeal exhibits enhanced sealing ability due to chemical interactions between calcium silicate and moisture within dentinal tubules, leading to the formation of hydroxyapatite. This may contribute to improved sealing of the root canal and reduce bacterial infiltration. Furthermore, the hydrophilic nature of the bioceramic sealer allows it to expand upon setting, forming a “self‐seal” [32].

The single‐cone obturation technique has gained increasing interest owing to its simplicity and efficiency, particularly when used with modern single‐file endodontic systems. It is also recommended that, when using coated gutta‐percha, the risk of coat debonding during obturation could be minimized [27, 33]. Although the single‐cone technique may be limited in adapting to the canal irregularities, particularly in the coronal and middle thirds, this limitation can be mitigated using bioceramic sealers. These sealers provide extensive surface contact and improved adaptation to both gutta‐percha and root dentin [34]. Accordingly, the single‐cone technique was selected in the present study to overcome the limitations of the cold lateral condensation technique, including its time‐consuming nature, lack of homogeneity of the obturation mass, and increased incidence of voids [35]. Previous studies have reported comparable marginal adaptation when bioceramic sealers are used with the single‐cone technique compared with lateral compaction, supporting its suitability for the in vitro evaluation of the interfacial sealing performance [36]. Similarly, a study by Kardon et al. [37] found no significant difference in sealing ability between the single‐cone and warm vertical compaction techniques. Based on these findings, the present study used the single‐cone technique to evaluate apical leakage of uncoated and PDA‐coated gutta‐percha.

Deposition of a PDA coating on the gutta‐percha surface was confirmed by the observed color change and by the complementary chemical and morphological analyses. ATR‐FTIR findings indicated that the characteristic structure of gutta‐percha was preserved after coating, a result attributed to the extremely thin PDA layer. Concurrently, additional functional groups associated with the detection of PDA, consistent with a previous study by Ferreira et al. [38] which reported the persistence of the “same main peaks” in gutta‐percha samples even after plasma surface treatment. ESEM and EDX analyses further supported the presence of a uniform PDA layer on the gutta‐percha surface. These findings confirm effective surface modification without altering the core material, providing a suitable basis for evaluating the influence of the PDA coating on interfacial behavior with the bioceramic sealer.

The bioactivity of the PDA‐coated gutta‐percha was assessed following immersion in SBF. ESEM observations revealed the formation of hemispherical deposits on the coated cones, accompanied by the detection of Ca and P elements by EDX analysis. Compared with the uncoated gutta‐percha, which showed no comparable changes following immersion. These findings indicate that the PDA‐coated surface promotes mineral deposition under laboratory conditions. When used in conjunction with a bioceramic sealer, such surface mineralization may enhance the interfacial interaction between the sealer and the PDA‐coated gutta‐percha.

Previous research has demonstrated that gutta‐percha surface modification with bioactive coatings, such as calcium silicate or bioglass, can enhance surface wettability and promote apatite deposition upon contact with body fluids [12, 39]. These findings support the underlying concept of the present study, which states that PDA coating was used as an interfacial modification strategy, in combination with a bioceramic sealer, to promote surface mineralization and improve sealer–PDA‐coated gutta‐percha interaction under laboratory conditions. The dye penetration method was selected in the present study due to its simplicity, availability, and suitability for the preliminary evaluation of sealing performance under standardized laboratory conditions [40–42]. Methylene blue was chosen for its cost‐effectiveness, high staining capability, ease of use, and molecular size, which closely resembles that of bacterial by‐products that can escape contaminated root canals and irritate periapical tissues [43].

According to the dye penetration results, roots obturated with PDA‐coated gutta‐percha exhibited significantly lower apical dye penetration compared with those obturated with uncoated gutta‐percha, both with the bioceramic sealer using the single‐cone technique. This reduction in dye penetration may be attributed to the enhanced sealing ability, wettability, and interfacial interactions provided by the PDA coating and the bioceramic sealer. These findings are consistent with previous studies demonstrating improved sealing performance following surface modification of gutta‐percha using bioactive or hydrophilic coatings [33, 44]. Although the underlying mechanisms differ, the results collectively emphasize the importance of optimizing the sealer–core interface to enhance sealing and minimize microleakage during single‐cone obturation techniques.

Since dye penetration tests do not distinguish leakage at the dentin–sealer or sealer–gutta‐percha interfaces, SEM analysis was performed to further evaluate interfacial adaptability. Adaptation was assessed in transverse root sections at the apical, middle, and coronal levels. The PDA‐coated gutta‐percha group demonstrated improved marginal adaptation compared with the uncoated group at all evaluated levels, with the greatest adaptation at the coronal level and the least at the apical level. This gradient may be related to variations in sealer thickness and distribution along the root canal. These findings are consistent with previous studies reporting superior adaptation in coronal regions compared with apical regions when using bioceramic sealers [45] and highlight the influence of canal anatomy and sealer volume on interfacial integrity. Conversely, in a study by Tay et al. [46], resin‐based sealers produced more gaps in the coronal region than in the apical region. This was attributed to the greater volume of sealer needed coronally, resulting in a more pronounced volumetric shrinkage. In the present study, the highest marginal adaptability values were observed for the PDA‐coated gutta‐percha with a bioceramic sealer after 28 days of SBF immersion, exceeding those at other time points within the same group and those of the uncoated gutta‐percha with a bioceramic sealer at all time points. This could be explained by the bioactivity and hydrophilicity of the PDA coat, which led to better adaptation and adhesion to the bioceramic sealer along the gutta‐percha surface.

Several studies have investigated strategies for modifying gutta‐percha surfaces to enhance bioactivity and wettability. Resin‐coated gutta‐percha cones used with hydrophilic methacrylate‐based sealers were originally introduced to chemically bond to the sealer and form a single “monoblock,” but subsequent studies reported incomplete and nondurable seals, partly attributable to polymerization shrinkage of the resin sealer and to debonding of the resin coat from the gutta‐percha core during handling [46]. Bioceramic‐coated gutta‐percha cones, such as calcium silicate‐impregnated cones, were similarly developed to promote biomineralization and enhance chemical interaction with bioceramic sealers; however, the coating layer is often nonhomogeneously distributed along the cone surface, which can compromise sealing and adaptation [47]. More recent experimental modifications, including bioceramic‐loaded and silver–mesoporous calcium silicate gutta‐percha, have shown improved bioactivity and apatite‐like deposition but have not consistently demonstrated superior interfacial adaptation across all root levels [48, 49]. These findings support the rationale for exploring alternative surface functionalization approaches.

PDA forms a thin, adherent organic interphase via dopamine self‐polymerization rather than depositing an inorganic mineral layer, and the resulting catechol‐ and amine‐rich surface increases surface energy, thereby improving wettability with the bioceramic sealer. Within the limits of the present in vitro design, this difference in coating chemistry may help explain the reduced apical dye penetration and the gap‐free interface observed on day 28 with the PDA‐coated cones.

Therefore, the null hypothesis was rejected as PDA‐coated gutta‐percha demonstrated significantly improved sealing ability, evidenced by reduced apical dye penetration; enhanced interfacial adaptability, as confirmed by SEM evaluation; and superior wetting ability, as indicated by lower contact angle measurements compared with uncoated gutta‐percha.

A major strength of this study is the use of multiple complementary evaluation methods, including the dye penetration test, adaptability test, and wetting ability test. These methods provide a comprehensive assessment of the sealing ability and interfacial adaptation of PDA‐coated gutta‐percha with the bioceramic sealer using the single‐cone technique.

From a clinical perspective, improving the interfacial adaptation between gutta‐percha and bioceramic sealers may enhance the long‐term sealing ability of root canal fillings. Importantly, the PDA coating forms a thin organic layer that does not alter gutta‐percha’s core properties and is expected to remain retrievable with conventional retreatment techniques, such as solvents and mechanical instrumentation. It is inexpensive and easily scalable due to its simple self‐polymerization process, supporting potential commercial translation.

The present study has several methodological limitations, including the potential introduction of artifacts during specimen sectioning for SEM analysis, such as microcracks or partial detachment at the gutta–percha interface, despite the use of water cooling, which may obscure the proper adaptation to the bioceramic sealer. Micro‐CT can eliminate sectioning artifacts and provide volumetric assessment; however, its current resolution and radiopacity overlap among materials still limit its sensitivity relative to SEM. Regarding sealing ability evaluation, the dye penetration method—while widely used and cost‐effective—has limitations compared with more advanced techniques such as fluid filtration, bacterial leakage models, or micro‐CT analysis [26, 50]. Nevertheless, when standardized protocols are applied, dye penetration remains a reproducible method for preliminary comparative in vitro assessment.

The present study has sample‐related limitations: the sample size for the sealing ability test was determined through prior power analysis and was sufficient to detect statistically significant differences; however, the relatively small number of specimens may limit the generalizability of the findings. The adaptability analysis was based on representative roots at each time point. The adaptability assessment was intended as a qualitative, descriptive analysis of the core–sealer interface rather than as a basis for statistical inference. Therefore, future studies with larger sample sizes and quantitative three‐dimensional analyses are needed to confirm and extend the present observations.

Several clinical limitations were presented in this study as it was limited to in vitro testing. Consequently, the findings may not directly reflect clinical performance. Further studies evaluating thermal cycling, long‐term durability, bonding stability, handling characteristics, and the clinical relevance of PDA‐coated gutta‐percha compared with other surface modification strategies are needed before clinical translation.

## 5. Conclusion

Within the limitations of this study, PDA‐coated gutta‐percha demonstrated improved in vitro sealing characteristics, including reduced apical dye penetration, improved sealer‐core adaptation, and enhanced wettability when used with a bioceramic sealer compared with uncoated gutta‐percha. An improved interfacial interaction between the sealer and gutta‐percha may contribute to more effective sealing under laboratory conditions. Further in vivo and long‐term studies are required to determine the clinical relevance and durability of this surface modification approach.

## Author Contributions

**Mariam Fahmy**: conceptualization, methodology, investigation, formal analysis, validation, visualization, writing – original draft, writing – review and editing, project administration. **Aiah A. El-Rashidy**: conceptualization, methodology, investigation, formal analysis, validation, resources, supervision, project administration, writing – review and editing. **Taheya Moussa**: conceptualization, methodology, formal analysis, resources, supervision, writing – review and editing.

## Funding

The authors received no financial support for the research and authorship of this article.

## Disclosure

All authors have read and approved the final version of the manuscript. The corresponding author (Mariam Fahmy) had full access to all data in this study and takes full responsibility for the data’s integrity and the accuracy of the analysis.

## Ethics Statement

Extracted human teeth in this study were used according to the approval of the Ethical Committee of the Faculty of Dentistry, Cairo University (Number 4‐9‐23).

## Conflicts of Interest

The authors declare no conflicts of interest.

## Data Availability

All the data generated or analyzed in this study are included in this article in the form of tables and figures. The raw data that support the findings of this study are available upon request from the corresponding author.
